# Imbricated Coastal Boulder Deposits are Formed by Storm Waves, and Can Preserve a Long-Term Storminess Record

**DOI:** 10.1038/s41598-019-47254-w

**Published:** 2019-07-25

**Authors:** Rónadh Cox, Louise O’Boyle, Jacob Cytrynbaum

**Affiliations:** 1 Department of Geosciences, Williams College, Williamstown, MA 01267 USA; 2 Earth Institute, University College Dublin, Belfield, Dublin 4 Ireland; 3 School of Natural and Built Environment, Queen’s University Belfast, Belfast, UK

**Keywords:** Geomorphology, Natural hazards, Physical oceanography

## Abstract

Coastal boulder deposits (CBD) are archives of extreme wave events. They are emplaced well above high tide, and may include megagravel clasts weighing tens or even hundreds of tonnes. But do they represent storms or tsunami? Many are interpreted as tsunami deposits based simply on clast size and inferences about transport, despite the fact that there are no direct observations documenting formation of these inbricated boulder clusters and ridges. In this study, we use force-balanced, dynamically scaled wave-tank experiments to model storm wave interactions with boulders, and show that storm waves can produce all the features of imbricated CBD. This means that CBD, even when containing megagravel, cannot be used as *de facto* tsunami indicators. On the contrary, CBD should be evaluated for inclusion in long-term storminess analysis.

## Introduction

**Figure 1:**
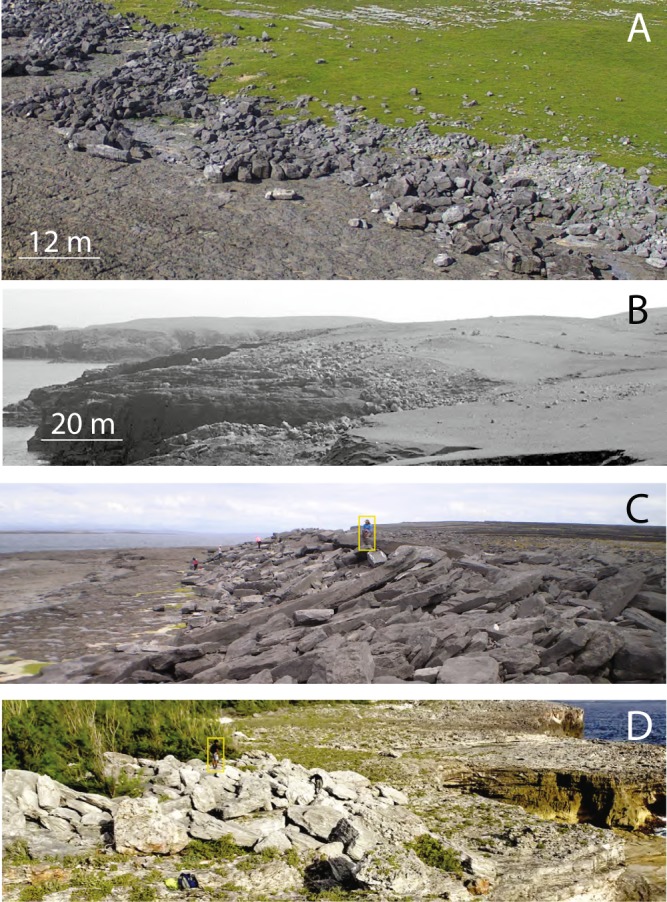
Examples of coastal boulder deposits (CBD), showing the difference between boulder clusters on unrestricted platforms and boulder ridges on backstopped platforms. (**A**,**B**) Show unrestricted platforms: the terrain is uneven, but there are no abrupt breaks in slope to focus boulder deposition. On unrestricted platforms, boulders form weakly organised clusters, which are low, wide, and irregular, with a poorly defined seaward edge. Imbrication is evident but not pronounced. In contrast, (**C**,**D**) are backstopped platforms: i.e. bedrock steps inhibit the movement of clasts and provide ridge-nucleation sites. The bedrock backstops are occluded by boulders in these images, but it’s clear that the ground level inland of the ridge is higher than the bedrock platform in front of the ridge. On backstopped platforms, CBD take the form of strongly imbricated, linear boulder ridges. The ridges are steep and relatively narrow, with a well-defined, abrupt seaward toe. Scale bars in (**A**,**B**) refer to the mid-ground of the image. Yellow boxes in (**C**,**D**) show people for scale. (**A**) Co. Clare, Ireland; (**B**) Villians of Hamnavoe, Shetland (photo by James Hansom, used with permission); (**C**) Inishmaan, Ireland; (**D**) Eleuthera, Bahamas.

**Figure 2:**
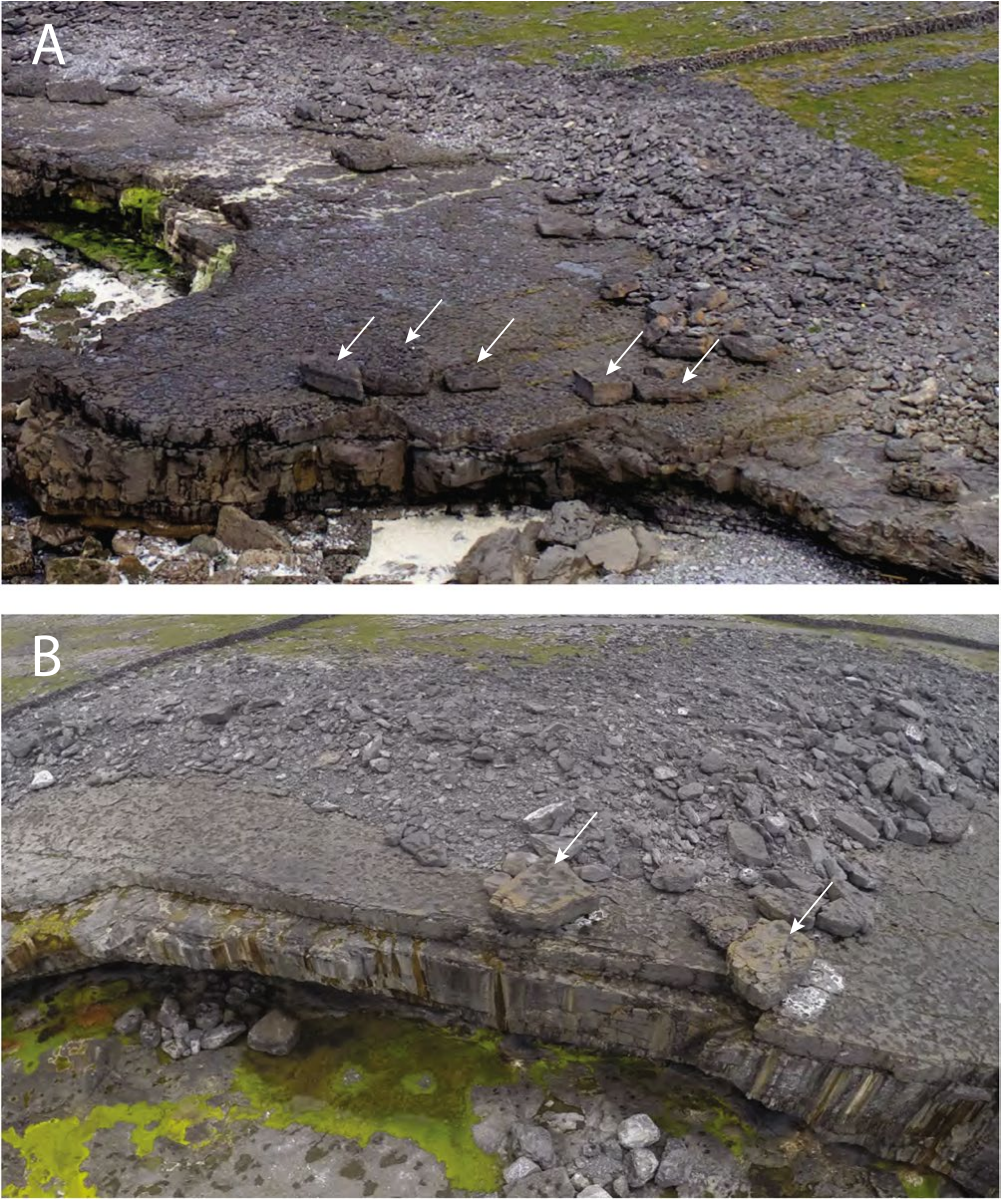
Cliff-edge boulders (shown by arrows) in association with boulder ridges on the Aran Islands. The lighter the colour, the fresher the surface. Pale colours of boulders in the ridges indicate recent reworking. The dark surfaces of the cliff-edge boulders suggest that they have been in that orientation for a considerable time. The boulders in (**A**) are those discussed in Erdmann *et al*.^11^; their Fig. 10. White patches to the right of the two cliff-edge boulders in (**B**) were exposed when those two boulders were shunted slightly to the left during the 2013–2014 winter storms. The white material is calcium carbonate precipitate, which forms beneath the boulders if they remain in one place for a long time. The position of the patches indicates that the block-moving waves came across the platform from right to left. Per the results of this study, waves approaching the cliff head on would have been unlikely to shift the boulders.

**Figure 3:**
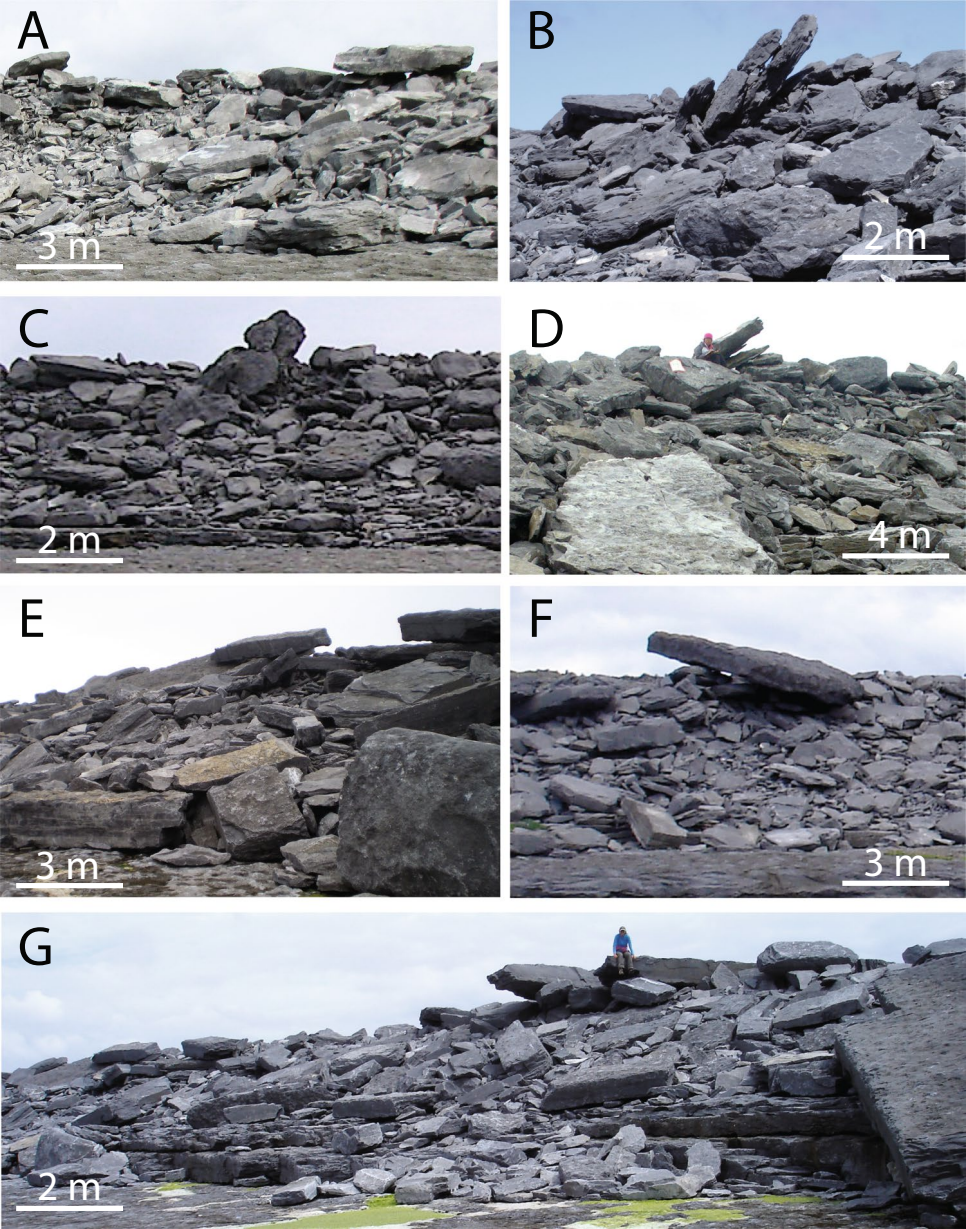
Examples (all from the Aran Islands, Ireland) of boulder ridges including crest-line clasts in unstable or delicately balanced positions. Boulders in such settings have been interpreted as a signature of tsunami emplacement^12^, but the clasts in D and G (with people sitting on them) were emplaced by storm waves in winter 2013–2014^1^, and results presented in this paper show that storm waves will routinely emplace balanced clasts. In all images, the view is landward and the ocean is behind the photographer. These deposits range from 10–20 m above high water, and the ridge bases are 50–220 m inland of the high-tide line.

Coastal boulder deposits (CBD) accumulate above high tide, at considerable distances inland in many cases. They commonly take the form of elongated boulder ridges or clusters, and can include megagravel weighing tens or hundreds of tonnes (Fig. 1). Many characteristic features of CBD have been promoted as signatures of a tsunami origin. Bryant^1^ lists clustering and imbrication as two of ten criteria indicating that CBD are emplacemed by tsunami, and further maintains that storm waves are unlikely to deposit large boulders in imbricated piles on cliff tops. Sustained flow, characteristic of tsunami, is given as a requirement for megagravel imbrication e.g.^2–4^, with suspension transport assumed in some cases^5^; leading to boulder ridges being interpreted as a signature of tsunami overwash^6^. Erdmann *et al*.^7^ speculate that weathered cliff-edge boulders (Fig. 2; see also^7^, their Fig. 10), which appear to have been unmoved for centuries despite being subject to the full energy of overtopping waves, must have been emplaced by tsunami in the distant past. Similarly, boulders in delicately balanced positions (Fig. 3; see also^8^; e.g. their Fig. 11) must signify tsunami, because (the argument goes) any large rock landing in an unsteady setting during a storm would be toppled and repositioned by subsequent waves. Thus the suite of CBD characteristics—large clast size, distance from shore, imbrication, and long-term stability of cliff-edge and balanced clasts—are asserted to signify tsunami flow^1,6,8–10^. These criteria have then been used as the basis for interpreting many CBD as tsunamigenic^2,4,11–19^.

A storm-wave origin for these same features, however, is advocated by many. Some emphasise the absence of any observations showing imbricated boulder ridges being formed by tsunami^20,21^. Others argue that well-developed imbrication in CBD requires transport and reorganisation by many waves, implying storms^20,22,23^. Recent observations show that storm waves are capable of moving the largest clasts^24–27^. And the sheer size and volume of some ridge complexes^22,28,29^ is interpreted to require repeated wave activity rather than a few tsunami^30–32^.

But arguments on both sides are largely inferential. Direct observations are few, and debate persists because of this data vacuum. The largest clasts move only during uncommon, extreme events, so most CBD accumulate and evolve over millennia e.g.^28,33–35^. And because interest in CBD is quite recent (A Google Scholar search using the terms “wave” “storm” “tsunami” “boulder” shows that about 90% of studies on this topic post-date 2000) the longitudinal studies and systematic data necessary to formulate robust criteria are lacking^30,36^. In particular, there are no records of the initiation and growth of imbricated boulder ridges.

It matters that we know whether boulder ridges are formed by storms or tsunami. Risk assessments are based on the history of extreme events, and this is greatly complicated if storm deposits are misinterpreted as tsunamigenic, or vice versa^32,37^. Field observations are critical; but because of the long timescales over which extreme events occur, theoretical approaches and experiments are also necessary^31^. Numerical models as yet lack the non-linear complexity to produce detailed and specific solutions^38^, so force-balanced wave-tank investigations offer the best near-term approach. Dynamically scaled physical experiments can expose model boulders to realistic storm conditions, preserving real-world complexity, including stochastic fluctuations and interactions. We can produce extreme events on demand, and duplicate centuries of activity in the space of a few weeks, providing synthetic coastal storm records that can be compared with CBD field data.

## Methods

This paper reports Froude-scaled wave-tank experiments testing whether storm waves can construct imbricated boulder ridges on a supratidal platform. Using the well-studied Aran Islands^23,24,28,39,40^ as the model prototype, we constructed a cliffed coast rising out of deep water, fronted by ramp-and-flat bathymetry. A cliff-top platform hosted limestone clasts. The platform was flat for some tests, dipped 3–7° seaward for others; and had unrestricted extent in some cases, versus a resistant backstop in others (see Detailed Methods). A synthetic storm with significant wave height (H_s_) of 12 m, computer coded based on a JONSWAP^41^ spectrum, was implemented in the tank via a suite of independently programmable wave generator paddles. The storm was run multiple times in each of twenty-one different setup tests. There were five to sixteen runs per test, for a total of 239 runs (See Detailed Methods, Table 1). Clast distributions were continuously recorded, documenting patterns of transport and deposition.

## Results

Scaled storm waves in these experiments reproduced all the characteristic features of CBD, including imbricated ridges and clusters, stranded cliff-edge boulders, and delicately balanced clasts.

**Figure 4:**
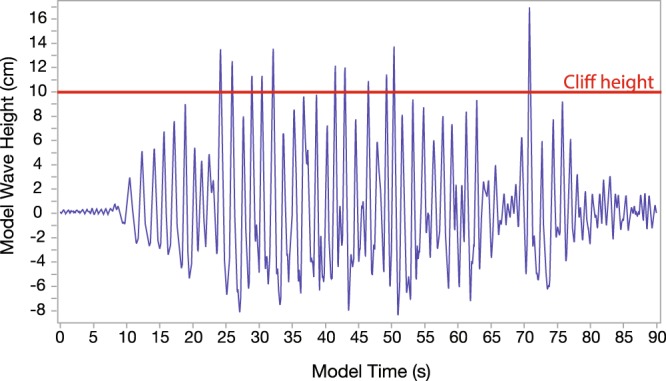
Wave trace of a single storm segment, recorded by the wave gauge positioned 8 cm from the model cliff (see Detailed Methods). The cliff height, 10 cm above the still water level, is indicated by the red line. The 90-second record includes several seconds of initial quiet water (allowed to calm between each run), followed by the 64-second storm segment, and then several seconds of energy tailing off at the end of the sequence. Wave heights in cm correspond to prototype wave heights in m. Time values on the X axis should be multiplied by10 to get prototype times in seconds. Note that the wave conditions at the gauge differ from the JONSWAP input spectrum, as they include evolution across the changing bathymetry, and also reflections from the cliff. See Movie 01.

Clasts were moved inland and against gravity, both updip (when the platform was tilted at 3° or 7°) and by stacking on top of other clasts. Each storm had relatively few clast-moving waves (Movie 1), as only twelve waves in each storm segment were large enough to overtop the cliff (Fig. 4). Overtopping waves generated cross-platform flows with durations in the tens of seconds (hundreds of seconds scaled equivalent), i.e. much greater than the initiating wave periods. Velocities for boulder-moving flows (measured from video footage) were 80–250 cm/s (8–25 m/s scaled equivalent), consistent with reported storm-wave-generated flow velocities e.g.^42,43^.

### Modes of transport and emplacement

Some waves, by a combination of strong initial impact and an energetic bore, caused a lot of clast motion. Others, overtopping more gently, produced less change (Movie 1). Only the smallest clasts were fully submerged during transport. Small clasts tumbled along, rolling or saltating. Larger clasts slid. Some were overturned, but after a single inversion were transported by sliding, in contact with the platform. Clast size was not an absolute predictor of transport: sometimes larger boulders moved while smaller ones remained stationary. Some clasts were carried seaward by backwash, and fell from the cliff into the ocean; but most travelled inland.

Clasts swept inland were crudely fractionated by size. Boulders continued to move until either they reached a point where flows were not competent to entrain them, or they were obstructed, becoming part of a cluster or ridge. Larger clasts moved shorter distances, most coming to rest 30–50 cm (30–50 m) from the cliff edge, forming a partial impediment to other clasts.

### Deposit formation: clusters and ridges

**Figure 5:**
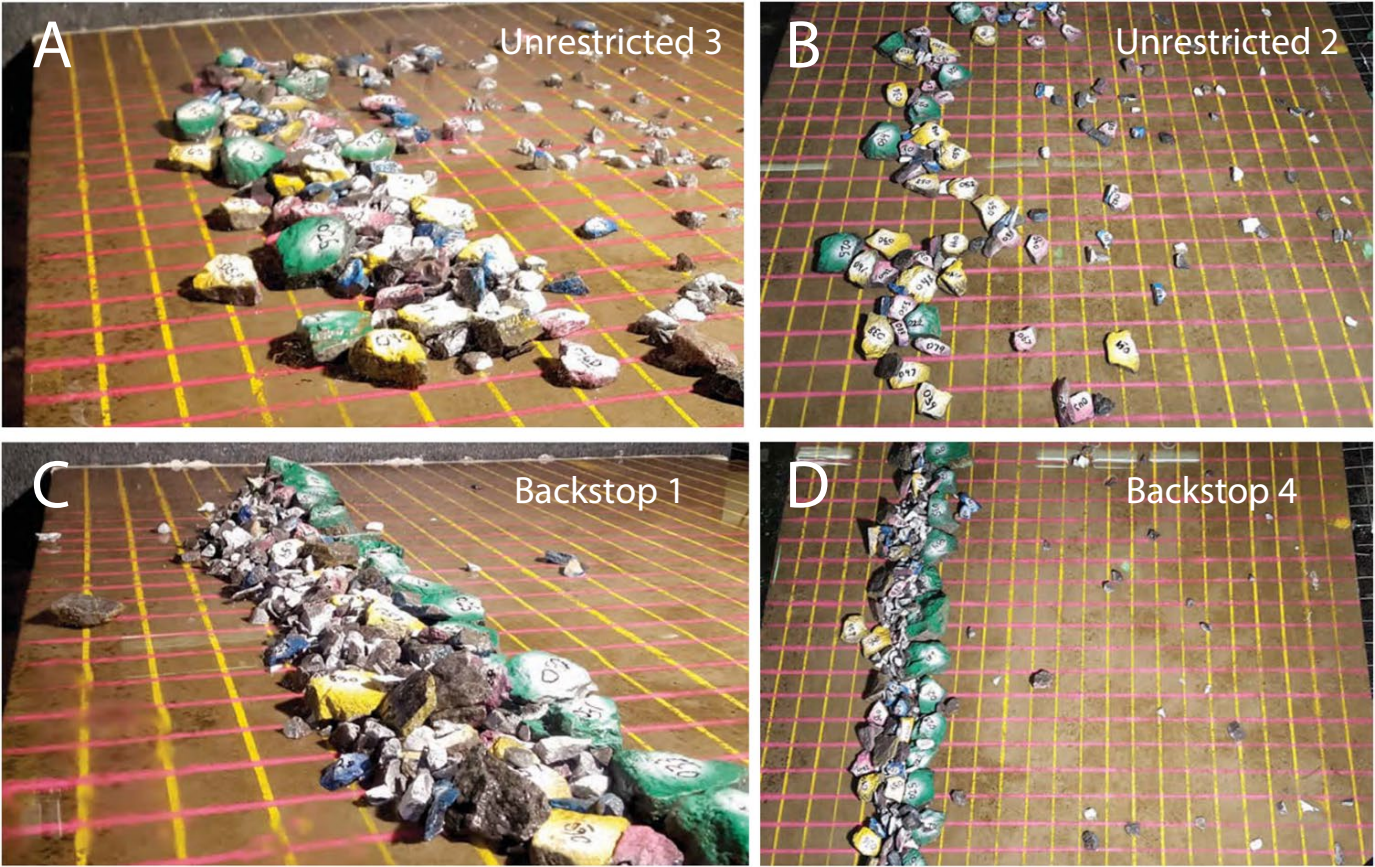
Representative deposit configurations. The specific tests (see Table 1) are indicated on each image. (**A**,**B)** Show unrestricted platforms and (**C**,**D**) show backstopped platforms, in which a line of the largest clasts was deployed to model a resistant but potentially erodible bedrock restriction. Grid spacing is 5 cm (=5 m at full scale), and clast colours and numbers are keyed to Supplementary Table S1.

Imbricated aggregations formed in every test (Fig. 5). Whether they were weakly organised clusters or well-defined boulder ridges depended on platform configuration. Where unrestricted, boulders clustered but tended not to stack more than a couple deep; whereas on backstopped platforms they formed higher, narrower, more linear ridges.

**Figure 6:**
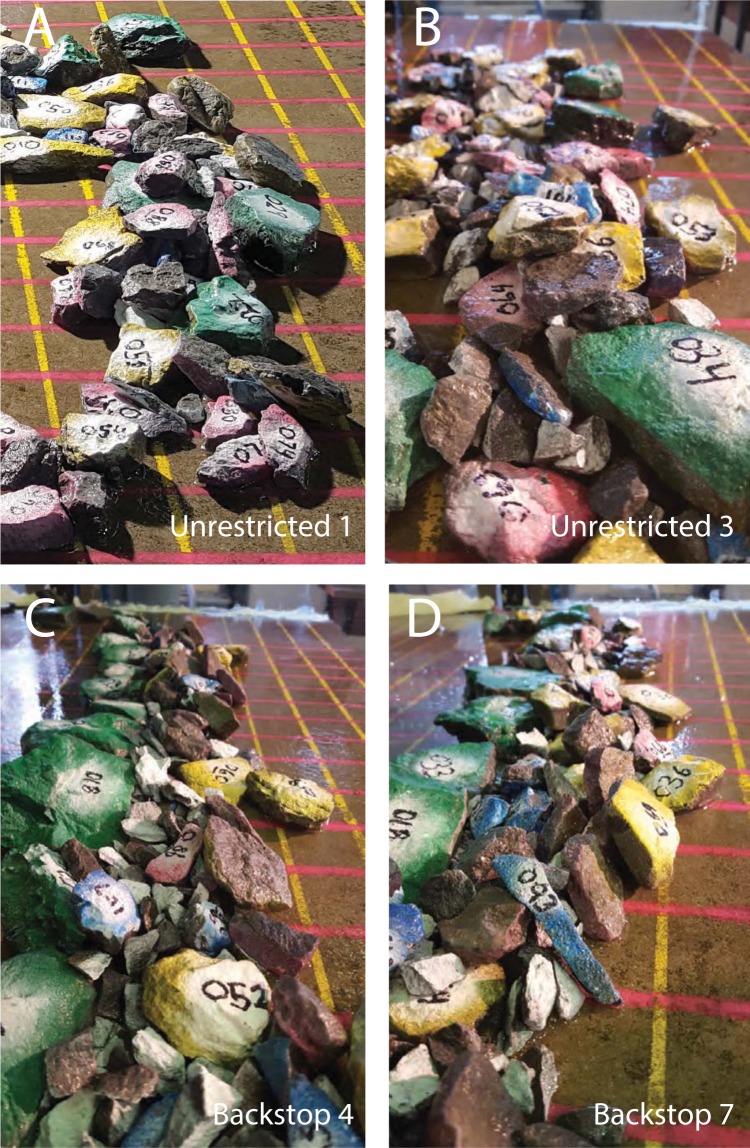
Patterns of stacking and imbrication from several different tests. The specific tests (see Table 1) are indicated on each image. (**A**,**B**) Show unrestricted platforms and (**C**,**D**) show backstopped platforms. Grid spacing is 5 cm (=5 m at full scale), and clast colours and numbers are keyed to Supplementary Table S1.

On unrestricted platforms, boulder clusters tended to have an irregular seaward margin, and variable cross-platform width (Fig. 5A,B). Stacked clasts were imbricated seaward; but because a relatively a small proportion of clasts became stacked, imbrication was not prominent (Fig. 6A,B). These features match those seen on unrestricted coastal platforms (Fig. 1A,B). Without a substantial obstruction to clast transport, there was little means for building depth in the deposit. Boulders migrated inland either by flow entrainment or by impulsion from other clasts (Movie 2). Larger boulders arrived at an equilibrium location (for the prevailing conditions) and accumulated there. Although there was substantial jostling and lateral motion of large clasts at the front of the deposit (Movie 3), the cluster core stayed in place. Some small clasts lodged in spaces between larger boulders, but many were transported around or over the deposit and were dispersed widely inland. The final pattern on unrestricted platforms was one or more clusters of abutting and overlapping boulders (mostly flat lying, some stacked or tilted), generally 5–10 clasts wide and 1–2 clasts high, grading to an inland-fining scattered boulder field (Figs 1B and 5).

On backstopped platforms, boulder ridges were linear and relatively uniform in their dimensions (Fig. 5C,D). The backstop restricted inland movement of the first boulders to arrive. Later-arriving clasts were driven up against the growing barrier. This resulted in clasts stacking, sometimes several deep (Fig. 6C,D). Many smaller clasts became trapped, and—relative to the unrestricted case–fewer travelled past the accumulation. The final pattern on backstopped platforms was a well-developed ridge, generally 4–6 clasts wide, and several clasts high. Within the ridge, most boulders were steeply imbricated, so that the deposit was narrow, and the footprint of each individual boulder relatively small. Some small clasts were dispersed further inland, but compared to the unrestricted case, these scattered boulder fields contained relatively few clasts.

Deposit width (defined as continuous platform cover, excluding scattered boulder fields) was 5–30 cm for clusters and 10–25 cm for ridges. Heights were 1.5–4 cm for clusters, and 3.5–7 cm for ridges (Figs 5 and 6). These dimensions, scaled up, match field measurements of CBD^22,28,44,45^, e.g.^46^.

### Boulders are stacked by being piled but also by being jacked up from below

Clast piles were built by two main mechanisms: flow traction (whereby a clast was lifted or overturned, landing on top of another clast) and jacking (one clast pushed beneath another, elevating the overlying clast). Traction transport was common for smaller clasts, which simply rolled or saltated on top of other clasts; and some large tabular clasts were overturned if their seaward edges were exposed to flow action. But the unexpected result was that jacking of large clasts was rather frequent: if a smaller clast (often much smaller) was inserted beneath the overhanging lip of another, successive waves often pushed it farther under, raising the overlying clast (Movie 3).

The most common kind of jacking was partial jacking followed by hydraulic overturning. In these cases, one or more small clasts became wedged under the seaward edge of a larger boulder, canting it inland. Uplifted edges, exposed to incoming waves, are vulnerable to upending^47^. Some clasts were therefore tipped vertical by subsequent flows, and remained in that orientation, supported by adjacent clasts (Movie 3, pink arrows). If later waves removed the wedging clasts (which happened in several instances), the standing stone was left with no evidence to indicate the emplacement mechanism. In other cases, the upended block overturned completely, landing on top of neighbouring boulders. Sometimes the overturning boulder displaced others, moving them further inland and upward in the growing ridge, in a domino effect (Movie 3, red arrows).

### It’s hard to move a boulder on the cliff edge

We were surprised to observe that boulders sitting at the cliff edge often failed to move, even as larger clasts, inland of the edge, were transported (Fig. 5C and Movie 1); so we investigated this in more detail using boulders in the 50–99 g range (50–99 t scaled equivalent; yellow colour category), which had moved readily under the same wave conditions in other tests. When placed at the very edge of the cliff, many of these clasts failed to move inland over the course of multiple storm cycles (Movie 4). Of the clasts that moved, the majority began by flipping upside down, and were then slid inland by later wave events. Once dislodged from the edge they progressed inland during subsequent flow events.

### Balanced boulders appear unstable, but are not

A number of clasts ended up in settings that looked rickety but were remarkably secure. Some perched or balanced boulders were emplaced by flow, but many—perhaps most—were levered into position by the jacking action of other clasts being wedged beneath them. These included vertical “standing stones”, such as the one jacked up in Movie 3, (see also near-vertical clasts in Fig. 6D) as well as boulders perched wobblingly on other boulders (see e.g. clast 093 in Fig. 6D, and three smaller clasts on the crest of the deposit in Fig. 6B, between clasts 034 and 064). These configurations often remained stable over numerous storm cycles.

## Discussion and Conclusions

These results—demonstrating that simulated storm waves produce realistic CBD, with a full range of characteristic features—dovetail with recent field observations of megagravel transport during large storms^24–26,48,49^ to produce concrete evidence that CBD are related to storm-wave activity. A key finding is that overtopping storm waves interacting with loose megagravel always form some kind of imbricated deposit (Fig. 5), and that the details of the geometry match boulder clusters seen on real-world unrestricted platforms (Fig. 1).

The results show, unequivocally, that tsunami are not required to form boulder ridges and clusters, and that it is incorrect to infer a tsunami origin based on imbrication or balanced clasts. Although tsunami may contribute to CBD growth and migration—and deposits of scattered megagravel are likely to be due to tsunami e.g.^50,51^—imbricated supratidal boulder ridges and clusters are not signatures of tsunami.

Although the experiments model a single setting—on top of a 10 m cliff — the results are general because once waves have overtopped, the hydrodynamic work is done by cross-platform flow. Waves of lesser height could initiate flows of similar force at lower elevation. Larger waves could do the same on higher cliffs. Thus storm-wave action should be considered highly likely —possibly even the default interpretation—when analysing and interpreting the evolution of imbricated CBD clusters and ridges.

It seems likely that many CBD have been misinterpreted as tsunami deposits: based on strong correlations between deposit age clusters and climate cycles, Marriner *et al*.^37^ concluded that about 90% of Mediterranean deposits attributed to tsunami should be re-considered as possible storm deposits. This study reinforces that conclusion. Thus, age data from imbricated CBD should be considered for integration into long-range storminess calculations. Equally importantly, in the absence of any direct evidence that tsunami flow can form imbricated boulder ridges^20,31^, they should probably be removed from tsunami recurrence models.

The most recent IPCC report^52^ emphasises that “*increased flooding and damage to infrastructure are critically important in sensitive environments such as small islands*, *low lying coasts and deltas*”. Marine flooding threats are exacerbated by the effects of energetic waves^53^. Thus, extreme storms pose greater risks as sea level rises, underscoring the need to understand their chronologies and return times. Imbricated coastal boulder deposits are a repository from which we may be able to extract the long-term recurrence of extreme storm waves.

## Detailed Methods

Dynamically scaled, force-balanced wave tank models provide insights into systems that are difficult to otherwise measure. In this case, we are able to examine processes that are not only challenging to observe as they are occurring live (because of obvious difficulties with responding in real time to extreme events at remote locations, and the additional problems of making direct measurements of work being done by exceedingly energetic storm waves), but also take place on decadal to millennial time scales. The number of cliff-overtopping, boulder-moving flows that we imposed in the course of each test might happen over centuries in the real world. Thus tank experiments yield information about the likely mechanisms of CBD evolution on long timescales, which can inform new hypotheses and underpin further study of these systems in nature.

### Aran islands as the model prototype

We built a 1:100 scale model with coastline and bathymetry based on the Aran Islands (Supplementary Fig. S1). We chose the Aran Islands as the model prototype because CBD there are well surveyed, the geography is such that wave approach is generally orthogonal to the coast^23,54^, and the near-shore bathymetry is well mapped^55,56^.

The Aran Islands form a linear array approximately perpendicular to the prevailing southwesterly wave-approach direction^54^, which is also the dominant imbrication direction in seaward faces of Aran Islands boulder ridges^23,28^. The well-stratified Carboniferous limestone that makes up the islands dips gently westward at 1–3°^57^, and breaks readily along bedding planes and orthogonal joint sets^58^, yielding boulders that tend to be approximately rectilinear (Fig. 2). Wave erosion on the Atlantic coasts has excavated bedding planes to form bedrock platforms, and exploited bedding-normal joint networks to produce steep cliff faces (Fig. 3). A subdued version of this topography continues offshore, in a series of submarine ramps and flats. The coastal topography of the Aran Islands ranges from gently-sloping low-elevation platforms to sheer 50 m cliffs^23,24,59^, but the 10 m cliff in our model is representative of many sections of the coastline.

The geometric simplicity of the model means that although the Aran Islands are the nominal prototype, results from these experiments are applicable to other sites where deep ocean water shoals across irregular bathymetry before meeting a cliffed coastline (with due attention to differences related to topography, bathymetry, or coastal irregularities). In addition, because the boulder movement is accomplished by platform-crossing bores, the results are generalisable also to uncliffed situations, or any setting where wave shoaling generates bores of this magnitude.

### Froude scaling

Froude scaling (i.e. ensuring that Froude numbers are the same at model and full scales) optimises dynamic similarity between model and real-world prototype, preserving the balance between inertia and gravity forces. Widely used in tank experiments e.g.^60–63^, it ensures that the wave physics in the model matches that at full size^64^, even over several orders of magnitude (i.e. including 1:100 geometric scale)^60^.

Froude scaling is also applicable to coarse bedload transport by water, as long as the bed material is geometrically scaled, the model clasts are >5 mm diameter, and the material density is the same as the prototype^65–67^. In this case, the Shields parameters of flow and the threshold Shields number for initiation of motion are the same in model and prototype. It is impossible to preserve both Froude and Reynolds similarity in wave tank experiments; but this can be ignored in experiments where the water is fully turbulent. Because in the case of full turbulence the effects of viscous forces are proportionally minimised; and —because drag coefficients become almost constant beyond a certain turbulence level—differences in Reynolds number between model and prototype have no dynamic effect^62,68^.

Because the geometric scale factor of our experiments was 1:100, the Froude number $(=\frac{U}{\sqrt{gD}})$ of the prototype and the model are the same when mass is scaled by 100^3^ (1 g at model scale is 1t at full scale), density is scaled by a factor of 1 (1 g/cm^3^ in the model is 1 g/cm^3^ at full scale) and time is scaled by √100 (1 s in the model sis 10 s at full scale).

### Physical model setup

The experiments were run in the Queens University Belfast wave tank (Supplementary Fig. S1), which is specially designed for nearshore and coastal marine research^69^. The water, deepest next to the wave generator (54 cm deep in our experiments), shallowed across a pair of ramps (sloping at 8° and 3°, respectively) with intervening flats^70^. The tank being 14 m long, the 1:100 geometric scale factor allowed us to model a prototype in which waves shoaled across a 1.4 km distance, arriving at a 10 m cliff rising out of 20 m deep water, with boulders sitting on a 120 m wide cliff-top platform.

### The boulders

Arrayed on the platform were limestone pebbles and cobbles serving as model boulders, numbered and colour-coded to make it easier to track them during tests (Supplementary Fig. S2). We used limestone clasts, collected on the Aran Islands, to maximise model similarity (Supplementary Table S1). Their weights ranged from 5–457 g (5–475 t scaled equivalent), to capture the behaviour of the large clasts that are characteristic of CBD around the world e.g.^10,22,31,32,44,71–^74; these were our focus because they are at the centre of the controversy about emplacement mechanism. Rock density, calculated via water displacement and clast weight, averaged 2.61 g/cm^3^. Using clasts of this size (and not smaller: see Froude Scaling section above) ensured that the transport dynamics were properly scaled.

### Platform configurations

We implemented two different platform configurations—unrestricted and backstopped (Supplementary Fig. S2)— to mimic the kinds of settings in which CBD occur. In the unrestricted configuration, the platform was open, with no pre-existing ridges or barriers to boulder motion. This is the case at a number of locations in western Ireland and Shetland (Fig. 1A,B), as well at sites in the Caribbean^30^, and the Mediterranean^75^. In the backstopped platform configuration, we placed a line of large clasts 30 cm (30 m scaled equivalent) from the cliff edge, to mimic the effect of a pre-existing ridge or bedrock step. This configuration (Fig. 1C,D) is common for CBD, as seen in the Aran Islands^23,28^, Australia^76^, Crete^19^, and Iceland^22^. In our tests, the barrier was constructed of large clasts, so that it was strong but also potentially erodible, as a natural barrier would be. We experimented with platform dips of 0°, 3°, and 7° in both configurations. All experiments began with boulders spread out in the area 0–30 m or 0–40 m from the cliff edge.

### Wave generation and measurement

The tank was equipped with a six-paddle Edinburgh Designs (www.edesign.co.uk) Paddle Ocean Wave Generator, using force-controlled wave making^77,78^ to simulate fully definable, replicable deep-ocean waves and sea states. The six sector-carrier paddles were individually programmable, making them capable of generating realistic sea states^79–81^. Software measured and corrected for tank edge effects as the experiment was running, and force-based wave absorption countered reflected wave buildup, ensuring that sea states remained stable^82,83^. Computer control also ensured that the same sea states were precisely reproduced again and again in successive experiments.

Conditions in the tank were recorded using wave gauge controllers (manufactured by Edinburgh Designs), which measured electrical resistance of the water between a pair of parallel rods. Thus—resistance being proportional to immersion depth—the gauges recorded water level changes (Fig. 4). Readout rate was 150/s, and calibration tests showed reported heights to be precise within a millimeter. A gauge 8 cm from the cliff (Supplementary Fig. S1) measured the height of waves just before cliff impact.

Synthetic storms for this experiment were based on a JONSWAP (Joint North Sea Wave Project) spectrum^41,84^, using a standard peak enhancement value γ of 3.3^85–87^. We input the spectral parameters to the Wave Synthesiser (www4.edesign.co.uk/product/wave-generating-software) software, which generated a random sea state based on those conditions, and sent it to the wave generator. Before beginning the experiments, the sea state was run in the tank and recorded at the wave gauges to test how closely it conformed to the target spectrum. An iterative tuning processes followed, where calibration factors were tweaked within the software until the sea state measured in the tank matched the target criteria. The end result in this case was a 64-second sea state (equivalent to 10.7 minutes of storm sea at full scale), a sample wave-trace of which is shown in Fig. 4.

The experiments reported here were run with sea states having significant wave height (H_s_) of 12 cm (12 m at fule scale) and peak period (Tp) of 1.55 s (15.5 seconds at full scale). Based on long-term buoy data from the west of Ireland^88^, 12 m H_s_ occurs, on average, more than once a year. Thus this is a an appropriate sea state to use in simulation of strong but not rare events at the Aran Islands. We used the same synthetic storm in every run, so there were no variations in the number, size, groupings, or phase speeds of the constituent waves from one run to the next, making the tests fully repeatable. The data reported in this paper are a subset of a larger study into wave-boulder interactions using a range of sea states and platform configurations^89^.

### Structure of tests

We report a total of twenty-one tests, each consisting of five to sixteen runs of the storm cycle (Table 1). The main series of fifteen primary tests focused on boulder ridge formation. Of these, four tested with an unrestricted platform, and eleven included a backstop. The secondary set of six tests examined the stability of cliff-edge boulders.

**Table 1:** List of tests, showing the number of storm segments run in each, the platform dip, and the conversion to storm time at full scale.

Test name	Number of 64 s storm segments	Platform dip angle	Total wave-exposure time (minutes)	Storm length at full scale (minutes)
Unrestricted 1	16	0	17.1	182
Unrestricted 2	18	0	19.2	205
Unrestricted 3	15	3	16.0	171
Unrestricted 4	14	7	14.9	159
Backstop 1	13	0	13.9	148
Backstop 2	15	0	16.0	171
Backstop 3	16	0	17.1	182
Backstop 4	16	0	17.1	182
Backstop 5	15	0	16.0	171
Backstop 6	15	0	16.0	171
Backstop 7	15	0	16.0	171
Backstop 8	14	3	14.9	159
Backstop 9	15	7	16.0	171
Backstop 10	14	0	14.9	159
Backstop 11	5	0	5.3	57
Cliff-edge 1	4	0	4.3	46
Cliff-edge 2	4	0	4.3	46
Cliff-edge 3	4	0	4.3	46
Cliff-edge 4	4	0	4.3	46
Cliff-edge 5	4	0	4.3	46
Cliff-edge 6	4	0	4.3	46

The primary tests began with a population of boulders arrayed on the cliff-top platform; and each test consisted of many (fourteen to eighteen) consecutive 64-second storm segments (Table 1). At full scale (time in the model being 10x faster) this equates to approximately three hours of extreme sea state exposure per test. Four of these tests were run with an unbackstopped platform, and ten with a backstop 30 cm (30 m) inland of the cliff edge. For one test, we began with a backstop, and then removed it after a few storm segments.

An additional six tests focused on the stability of cliff-edge boulders (Table 1). We added these tests because in the course of our experiments, we noted that clasts positioned exactly at the cliff edge were less likely to move than, so to investigate this phenomenon we ran a series in which we positioned ten clasts exactly at the cliff edge (Movie 4). These tests each consisted of four 64-second storm segments (43 minutes scaled equivalent). We used boulders in the 50–99 g range (50–99 t scaled equivalent; yellow colour category), which had moved readily under the same wave conditions when placed in locations away from the cliff. They were a range of shapes, and we changed their orientations with respect to the waves in different runs.

During tests, we stopped the wave generator after every storm segment, to photograph the boulder distribution and allow the tank to calm (so that residual waves did not affect the next storm segment). The experiments in progress were recorded using both an overhead GoPro video camera (e.g. Movie 1), and side-looking 100 fps images taken through the glass wall of the tank. Velocities of platform-crossing flows were measured using the 100 fps footage. We documented evolving boulder arrangements using still photographs taken after every storm segment.

## Supplementary information

Movie 1

Movie 2

Movie 3

Movie 4

Supplementary Information

## Data Availability

The data in this study consist of the images taken during the wave-tank runs. Analysed images are available from the corresponding author on reasonable request.
