# Supplementary material for: Imbricated Coastal Boulder Deposits are Formed by Storm Waves, and Can Preserve a Long-Term Storminess Record
Source: Sci Rep. 2019 Jul 25;9:10784. doi: 10.1038/s41598-019-47254-w (PMC6658525; doi:10.1038/s41598-019-47254-w)
Supplement: Supplementary file 5 — Supplementary Information [file 41598_2019_47254_MOESM5_ESM.pdf]

Imbricated coastal boulder deposits are formed by storm waves, and can preserve a long-term storminess record

Rónadh Cox, Louise O'Boyle, and Jacob Cytrynbaum

Supplementary information:

Includes
Captions for Movie 1, Movie 2, Movie 3, and Movie 4
Supplementary Figure S1
Supplementary Figure S2
Supplementary Table S1

Movie 1 GoPro footage showing the first storm segment run in test “Unrestricted 2” (there were fifteen subsequent runs in this test: Table 1). The thin metal rod extending into the water near the cliff (middle of the field of view) is the wave gauge at which the wave trace in Fig. 4 was recorded. Note the relatively small number of cliff-overtopping waves, and also the size-independent stability of boulders positioned right at the cliff edge.

Movie 2 Sequence of GoPro stills taken at the end of each of the fifteen runs during test “Unrestricted 3” (Table 1), showing the progressive evolution of the deposit. Note that after several runs the core of the main cluster reaches an equilibrium configuration. Small clasts continue to move through and over the deposit, and the scattered boulder field grows; but the main cluster stops migrating inland (larger storms might reactivate it). At the seaward edge of the cluster, flow is competent to move some of the large clasts, which are transported laterally.

Movie 3 GIF generated from stills taken after each run during test “Backstop 2”. The closeups were taken to document boulder configurations at the end of each storm segment. The sequence highlights some of the different modes by which boulders were stacked within the deposit. Pink arrows show how partial insertion of a small dark (i.e. overturned) clast beneath yellow boulder 010 facilitated uplift of the seaward edge, leading to 010 being tilted vertical. The wedging clast was subsequently transported onto the ridge, leaving 010 balanced at the ridge edge. The yellow arrow tracks the simple overturning of yellow clast 054, which becomes stacked on other clasts. And the red arrows track yellow clast 04, which becomes uncovered half-way through the series. A small grey clast wedges beneath 04, causing it to overturn onto other clasts in the ridge, both narrowing and steepening the deposit. The action of the large overturning clast causes the superjacent small blue clast to also overturn, migrating upward and inward.

Movie 4 GoPro movie showing the first storm segment test “Cliff Edge 6” (Table 1). The yellow clasts used in these tests all moved readily during the main series tests, when their initial positions were further inland. This test included a further three storm segments, at the end of which the two overturned clasts had reached approximately 55 cm and 60 cm (=55 m and 60 m) inland; one other clast had been transported 50 cm (without overturning); four clasts had been dislodged and fell into the ocean; and three clasts remained unmoved on the cliff edge. Similar results were seen in other tests.

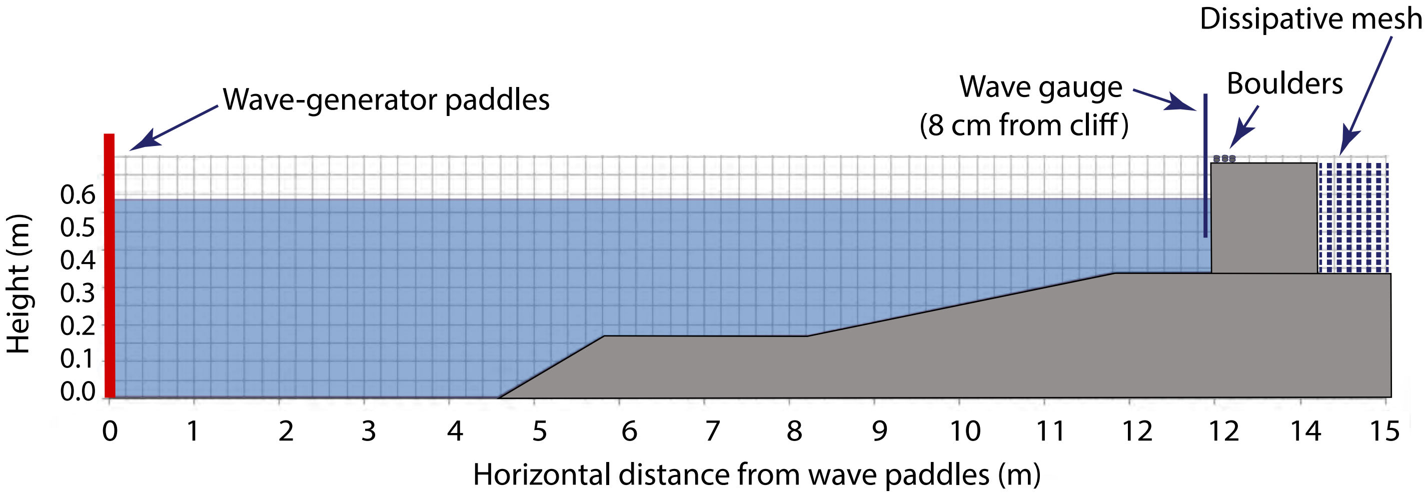

Supplementary Figure S1
Cross-section of wave tank setup, using measurements of Ward[89], showing configuration used in this study. Note the 4x vertical exaggeration of the Y axis. Water was 54 cm deep (54 m prototype equivalent) in the deepest part of the tank, adjacent to the wave generator, and shallowed progressively across ramps and flats to a near-shore depth of 20 cm (=20 m) adjacent to the cliff. The cliff rose 10 cm (=10 m) above the still-water level. Model boulders were arrayed on the cliff-top platform, which extended 120 cm (=120 m) from the cliff edge. The platform was level for some tests, and sloped seaward (at 3°-7°) for others, as is the case on the Aran Islands. A dissipative mesh behind the platform ensured that overtopping flow was not reflected back; backwash was driven only by gravity.

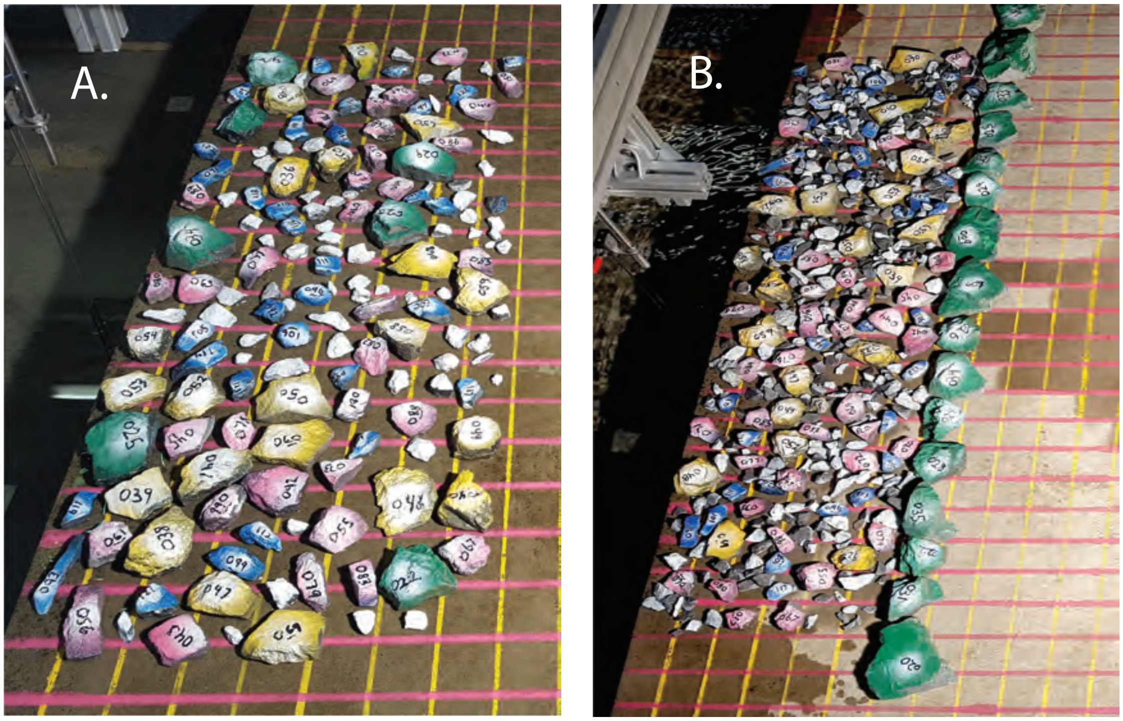

Supplementary Figure S2
Boulder population setup for (A) unrestricted and (B) backstopped platforms. The 5 cm grid on the platform top represents 5 m at full scale. Clast numbers and colours correspond to Supplementary Table S2

Supplementary Table S1. Catalogue of model boulders used in tests. Individual entries for clasts 10-500g. The smallest clasts (5-10 g) were not weighed individually and are not included in this catalogue. ID numbers and colours in Figs. 5, 6, and 8 correspond to entries in this table. Only the upper surface was painted, so clasts appearing grey in photographs were overturned in transport.

ID number	Colour	Mass (g)	X axis (cm)	Y axis (cm)	C axis (cm)
018	green	475	10.9	7.7	3.3
019	green	317	9.2	7.3	2.7
020	green	507	9.5	7.7	3.2
021	green	168	5.5	5.2	3
022	green	108	5.5	5.3	2.3
023	green	141	5.2	4.7	2.8
024	green	102	6.3	3.4	2.3
025	green	156	7.4	5.3	1.9
026	green	112	6.3	6	1.8
027	green	203	6.8	5.6	2.9
028	green	135	6.5	6	2.5
029	green	138	6.3	4.9	2.3
030	green	257	7.2	5.5	3.9
031	green	154	5.9	5.2	2.9
032	green	198	6.1	5.3	3.8
033	green	191	7.3	6.8	2.1
034	green	243	8.5	4.9	3.4
035	green	172	8.7	6.1	1.8
036	yellow	79	5.8	3.9	1.9
037	yellow	69	5.4	4.1	1.8
038	yellow	84	6.6	4.4	2
039	yellow	60	4.8	3.8	1.7
040	yellow	88	5.3	3.7	2.5
041	yellow	74	6.2	4.2	1.6
042	pink	49	4.4	4.3	1.3
043	pink	38	3.6	3.5	1.5
044	pink	40	4.2	3.7	1.1
045	pink	36	4.4	3.2	1.3
046	yellow	71	6.4	6	1.4
047	yellow	60	5.5	3.6	1.7
048	yellow	82	5.9	5.3	1.8

ID number	Colour	Mass (g)	X axis (cm)	Y axis (cm)	C axis (cm)
049	yellow	62	4.7	4.5	1.6
050	yellow	75	6.5	5.1	1.4
051	pink	32	3.7	2.2	1.8
052	yellow	57	4.9	4.1	1.5
053	yellow	56	5.3	3.3	1.4
054	yellow	68	4.3	3.5	2.5
055	pink	44	4.1	3.8	1.6
056	pink	36	6.8	2.7	1.1
057	yellow	66	6	3.4	1.6
058	yellow	74	5	4	1.8
059	yellow	76	5.9	4.4	1.8
060	pink	32	4.1	3.4	1.3
061	pink	33	3.5	3.4	1.4
062	yellow	75	4.7	4.7	1.4
063	pink	32	3.8	3.2	1.2
064	pink	31	4.3	2.7	1.1
065	pink	31	3.7	3.4	1.6
066	pink	34	4	2.6	1.7
067	pink	32	3.6	3.4	1.5
068	yellow	76	5.5	4.5	1.7
069	yellow	73	5.5	4.2	1.6
070	pink	22	3.6	3.1	1.1
071	pink	25	4.7	2.8	1.1
072	pink	23	4.8	3.7	0.8
073	pink	21	2.5	2.1	1.4
074	pink	21	4.7	2.9	0.8
075	pink	22	2.8	2	1.9
076	pink	24	3.4	2.4	1.6
077	pink	25	3.8	2.1	1.6
078	pink	24	3.6	2.4	1.4
079	pink	25	5.4	2	1.3
080	pink	22	3.2	2.5	1.2
081	pink	22	3.4	2.3	1.5
082	pink	23	3.9	3.3	1.2
083	pink	24	3.6	1.8	1.7
084	pink	22	3.6	3.4	0.9
085	pink	22	3.7	2.9	1.1
086	pink	25	4.8	1.8	1.5
087	pink	26	3.6	3.2	1.7
088	pink	23	3.8	3.6	0.8

ID number	Colour	Mass (g)	X axis (cm)	Y axis (cm)	C axis (cm)
089	pink	28	3.1	3	1.9
090	blue	12	3.2	1.9	0.8
091	blue	20	4.9	1.8	1.1
092	blue	18	3	2.1	1.4
093	blue	18	7.6	1.2	1
094	blue	15	3.1	2.5	1
095	blue	11	2.5	2.1	1.2
096	blue	12	3.1	2.3	1.1
097	blue	13	2.7	2.2	1.4
098	blue	17	3	1.5	1.3
099	blue	19	4.6	2.4	1
100	blue	20	3.8	2.4	1.4
101	blue	20	4.7	1.8	1.2
102	blue	14	3	2	1.1
103	blue	18	2.7	2.3	1.5
104	blue	17	3.5	2.4	1
105	blue	15	3.8	1.4	1.2
106	blue	16	3.1	2.6	0.9
107	blue	15	3.1	2.8	0.9
108	blue	17	2.8	2.3	1.5
109	blue	11	2.7	1.4	1.2
110	blue	13	2.9	1.9	1.3
111	blue	11	2.7	2.2	0.8
112	blue	11	3.3	1.5	1.2
113	blue	14	2.8	1.8	1.3
114	blue	19	4.8	2.8	0.7
115	blue	14	3.1	2	1.2
116	blue	11	3	2.4	0.9
117	blue	13	3	2	1
118	blue	12	3.9	2	0.8
119	blue	14	3.4	1.5	1.3
120	blue	14	3.4	2	1
121	blue	19	2.5	1.9	1.6
122	blue	20	3.3	2.6	1.1
